# Meta-analysis reveals profound responses of plant traits to glacial CO_2_ levels

**DOI:** 10.1002/ece3.836

**Published:** 2013-10-18

**Authors:** A A Temme, W K Cornwell, J H C Cornelissen, R Aerts

**Affiliations:** Department of Ecological Science, VU University AmsterdamDe Boelelaan 1085, 1081 HV, Amsterdam, The Netherlands

**Keywords:** CO_2_, glacial, growth, meta-analysis, photosynthesis, plant traits, subambient CO_2_

## Abstract

A general understanding of the links between atmospheric CO_2_ concentration and the functioning of the terrestrial biosphere requires not only an understanding of plant trait responses to the ongoing transition to higher CO_2_ but also the legacy effects of past low CO_2_. An interesting question is whether the transition from current to higher CO_2_ can be thought of as a continuation of the past trajectory of low to current CO_2_ levels. Determining this trajectory requires quantifying the effect sizes of plant response to low CO_2_. We performed a meta-analysis of low CO_2_ growth experiments on 34 studies with 54 species. We quantified how plant traits vary at reduced CO_2_ levels and whether C_3_ versus C_4_ and woody versus herbaceous plant species respond differently. At low CO_2_, plant functioning changed drastically: on average across all species, a 50% reduction in current atmospheric CO_2_ reduced net photosynthesis by 38%; increased stomatal conductance by 60% and decreased intrinsic water use efficiency by 48%. Total plant dry biomass decreased by 47%, while specific leaf area increased by 17%. Plant types responded similarly: the only significant differences being no increase in SLA for C_4_ species and a 16% smaller decrease in biomass for woody C_3_ species at glacial CO_2_. Quantitative comparison of low CO_2_ effect sizes to those from high CO_2_ studies showed that the magnitude of response of stomatal conductance, water use efficiency and SLA to increased CO_2_ can be thought of as continued shifts along the same line. However, net photosynthesis and dry weight responses to low CO_2_ were greater in magnitude than to high CO_2_. Understanding the causes for this discrepancy can lead to a general understanding of the links between atmospheric CO_2_ and plant responses with relevance for both the past and the future.

## Introduction

Atmospheric CO_2_ concentration has varied tremendously over geological time, from as high as 3000 ppm in the lower Devonian (Royer 2006) to as low as 180–280 ppm during the past 2.1 Ma of the Pleistocene (Honisch et al. 2012). About 17.5 Ka ago, atmospheric CO_2_ concentration started to rise from 180 ppm, levelled off at 280 ppm around 15 Ka ago and broadly remained at 280 ppm until the Industrial Revolution. Since the start of the Industrial Revolution, CO_2_ levels have risen to 390 ppm today, levels not experienced by plants for over 25 Ma (Royer 2006) and are expected to increase even further; common model estimates go up to 700 ppm by 2100 (IPCC 1985). A CO_2_ atmosphere of 700 ppm has not been observed since 42 million years ago (Royer 2006). The atmosphere today and as predicted for the end of the century is thus increasingly different from that experienced by plants during a large part of the recent past.

CO_2_ plays a pivotal role in a number of important ecophysiological processes: it is an essential ingredient for photosynthesis and plant growth, and it is highly likely that plants' morphological and physiological traits and their plastic responses to the CO_2_ concentrations are more tuned to the range of CO_2_ concentrations they have experienced recently. Because adjustment to changing CO_2_ involves changes in photosynthetic rates, nitrogen allocation, and other physiological properties (Curtis and Wang 2001; Korner 2007; Cowling 1987; Poorter and Navas 1988; Ainsworth and Long 2005), this trait adjustment has the potential to create a feedback that could affect the global carbon cycle (Beerling et al. 2007). Connecting the performance of plants of different species at low, ambient and future high CO_2_ is thus an important part of understanding the links between the atmospheric CO_2_ concentrations and the terrestrial biosphere in the past, present, and future.

At low CO_2,_ photosynthesis is limited by the amount of available carbon by limiting carboxylase activity of the enzyme RuBisCO. Conversely, at higher CO_2_ concentrations, the rate at which CO_2_ can be taken up (photosynthetic capacity) becomes limiting (Sage 2011). CO_2_ levels in the past could thus have selected for RuBisCO carboxylase activity or efficiency. At current and future CO_2_ concentrations, other factors such as water and nutrient uptake will become more limiting than CO_2_ (Ward et al. 1995; Campbell and Sage 2012; Reich et al. 2009). This implies that trait states useful in a low CO_2_ atmosphere can be redundant or suboptimal in a high CO_2_ atmosphere. For example, high investment in RuBisCO, useful at low CO_2_, in a high CO_2_ environment requires a high N investment, which could otherwise be used in other N-limited steps. High activity of enzymes facilitating transport and binding of CO_2_ is a lower priority at high CO_2_ compared with the need for more sink capacity of photosynthates to take advantage of increased photosynthetic rates (Sage and Coleman 1994). Favorable traits in low CO_2_ thus do not necessarily mirror those in high CO_2_.

For obvious reasons, considerable scientific effort has gone into examining the response of plants to high levels of CO_2_ as projected for the latter half of this century. Several recent meta-analyses have found that, despite methodological differences among studies, a few main results are apparent: at high (500+ ppm) CO_2,_ there is an increase in carbon assimilation and growth and decrease in stomatal conductance, nitrogen content and specific leaf area (Poorter 2010; Curtis and Wang 2001; Poorter and Navas 1988). The increase in biomass is about +45% for C_3_ species and +12% for C_4_ species at a 50% increase in CO_2_ concentration (Poorter and Navas 1988). The response of C_4_ species to increased CO_2_ is smaller than that of C_3_ species, probably because the carbon concentrating mechanism of C_4_ plants already concentrates CO_2_ around RuBisCO leaving less room for increased photosynthetic rate (Bowes 1985; Ghannoum et al. 2010). Overall, woody species showed a greater response to elevated CO_2_ than herbaceous species (Curtis and Wang 2001; Poorter and Navas 1988; Ainsworth and Long 2005; Lee et al. 2000).

In contrast to the large amount of studies on plant responses to elevated CO_2_, less research has been carried out on the response of plants to subambient, Pleistocene levels of CO_2._ Several individual experiments reveal that the influence of low CO_2_ acts on multiple biotic levels, ranging from leaf level to plant level and ecosystem level (Gerhart and Ward 1980). The emergence of agriculture has even been linked to the increase in CO_2_ to 280 ppm 17.5 Ka ago, as higher levels of CO_2_ lead to higher yields (Sage 2006). Understanding how plants have adapted to the low CO_2_ of their recent evolutionary history can aid us in understanding plants response to future high CO_2_ (Gerhart and Ward 1980; Beerling 2005; Leakey and Lau 2010). Recent research has shown CO_2_ uptake and water use are highly consistent across CO_2_, from low to high (Franks et al. 1982) Thus, there is clearly a need to integrate the knowledge available so far on low CO_2_ responses to determine whether more traits follow a predictable pattern.

Some qualitative expectations can be made as to how plants are likely to respond to low CO_2_. A lowering of CO_2_ will likely lead to a reduction in photosynthetic rates (Farquhar et al. 1995) and plant biomass (Overdieck et al. 2005; Cunniff et al. 1999). Next, an increase in leaf nitrogen concentration as RuBisCO may ameliorate some of the reduction in total C assimilation rate (Sage and Coleman 1994). Differences in response among different plant types can also be expected. C_4_ metabolism, which concentrates CO_2_ around RuBisCO, could partly compensate the potential reduction in growth as experienced by C_3_ plants. At lower atmospheric CO_2_ concentration, one of the mechanisms to maintain a high enough internal CO_2_ concentration is to open stomata wider, allowing water to escape at a faster rate (Farquhar and Sharkey 1998). Because of the carbon concentrating mechanism in C_4_ species, the diffusion gradient of CO_2_ across the stomata can be much steeper. At reduced CO_2,_ this should allow C_4_ plants to maintain a smaller stomatal aperture than C_3_ plants, giving a smaller increase in stomatal conductance and a smaller decrease in water use efficiency (Farquhar and Sharkey 1998). As woody plants invest more biomass in stems than herbaceous plants (Poorter et al. 2003) and as stems usually do not contribute substantially to photosynthesis, it is possible that this constrains the ability to adjust carbon allocation at low atmospheric CO_2_. In response to a reduction in growth, a complex suite of trait adjustments, with differences among plant types, is expected at all physiological levels, varying from photosynthesis to biomass allocation. While the directions of all these responses to low CO_2_ have empirical support (Gerhart and Ward 1980), they have not yet been quantified in general terms across studies and species.

Here, we present the results of a global meta-analysis synthesizing data from currently available low CO_2_ experiments to quantify general patterns of morphological and ecophysiological trait responses to subambient CO_2_. In particular, together with the body of work on high CO_2_, we aim to build toward a general, quantitative understanding of the response of plant traits to a range of CO_2_.

Thus, we address the following research questions and hypotheses:

1. How much do plant traits vary with decreased CO_2_ concentration?
  We hypothesize that lower photosynthetic rates will lead to reduced growth at low CO_2_. To acclimate to a low CO_2_ environment and keep up photosynthetic rates, plants will have higher leaf nitrogen and larger stomatal conductance.
2. How much do plant functional groups differ in their response to low CO_2_?
  Because of the carbon concentrating mechanism of C_4_ plants, we hypothesize (a) that the negative effects of low CO_2_ on their photosynthesis and growth will be reduced as compared to those in C_3_ plants; and (b) that woody species will invest more of their biomass in nonphotosynthetic tissue leading to a greater reduction in biomass accumulation than herbaceous species.
3. Is plant trait response to low CO_2_ similar in magnitude to the response to elevated CO_2_?
  Atmospheric CO_2_ is on a trajectory from low during glacial times to very high CO_2_ in the future. We aim to shed light on whether plant traits adjust similarly from low to ambient as from ambient to high. Given the saturating nature of the photosynthetic response to CO_2,_ we expect photosynthetic traits to respond to low CO_2_ through a greater magnitude shift compared with the high CO_2_ response. For other traits, we are curious if they follow the saturating response of photosynthesis or if they respond more proportional to CO_2_ changes.

## Methods

We performed a literature review on plant science journals searching Web of Science using keywords “subambient CO_2_,” “low CO_2_,” “reduced CO_2_” and “glacial CO_2_.” This resulted in 33 papers that reported on studies with experimentally lowered atmospheric CO_2_ concentration for 54 species in total. In these experiments, plants were grown in greenhouses, climate chambers, or outdoor tubes after Mayeux et al. (2010). For the climate chambers and greenhouses, CO_2_ concentrations were reduced by passing air through some kind of filter or adsorbent (e.g., Soda lime) or through a reactive solution (e.g., NaOH).

For each study, we recorded sample size, duration, growing conditions, low CO_2_ treatment type, and germination conditions along with measures of plant physiological traits at the CO_2_ concentrations used (Table S1, Data S1 and S2). A full list of the studies found with which species and trait data they reported can be found in Table S1, Data S1 and S2. When data and errors were not present in tabular form, they were extracted from graphs using Datathief 3 (Tummers 2006). All papers reported trait means in response to CO_2_ concentration and most reported a measure of error (standard deviation, standard error, or confidence interval) for the trait in each CO_2_ treatment. Three of the published papers dealt with response to low CO_2_ at varying resource conditions (P limitation: Campbell and Sage 2012; Lewis et al. 2012; Drought: Ward et al. 1995). In order to exclude confounding factors, only those results at high nutrients and well-watered conditions were included. C_3_–C_4_ intermediates were grouped together with C_4_ species.

From the studies found, only 6 traits emerged with 10 or more species analyzed and only 20 traits with 3 or more species. Of these 20 traits, 12 were related to growth and development. These traits included specific leaf area (as SLA or leaf mass per area, which was recalculated to SLA, m^2^·g^−1^) and (components of) plant biomass (DW, g dry weight). In some cases, plant biomass was divided into above- and belowground mass. Aboveground mass was divided into leaf and stem mass. All of these masses can be expressed either in absolute terms or as allocation, that is, relative to plant mass. Number of stomata and stomatal pore size (μm) were infrequently reported. Five traits related to photosynthesis included photosynthetic rate either as maximum, at saturating light levels (*A*_max_, μmol·m^−2^·s^−1^), and/or net, at growth conditions, photosynthesis (*A*_net_, μmol·m^−2^·s^−1^), stomatal conductance (*g*_*s*_*,* mol·m^−2^·s^−1^), the ratio of internal to external CO_2_ concentration (*C*_*i*_/*C*_*a*_) and water use efficiency (WUE; *A*_net_/*g*_*s*_, mmol·mol^−1^). Lastly, four traits were related to chemical composition, namely nitrogen content either area-based (g·m^−2^) or mass-based (g·g^−1^), chlorophyll content (μmol·g^−1^) and RuBisCO content (g·m^−2^).

To examine the effect of CO_2_ among all species in the study, we performed a weighted ANCOVA for each trait with CO_2_ concentration as covariate, species as a factor, and a potential interaction between the two. To determine the overall effect of CO_2_ on a plant trait, a model without differing slopes between species was fitted when species by CO_2_ interaction was not significant or the average slope from all species was calculated when species by CO_2_ interactions were significant. We used the inverse of the square root of the standard error from the original study as the weighting factor for uncertainty, as is standard in meta-analyses (Hedges and Olkin 2000; Sokal and Rohlf 1992) For trait values reported without an error term, the average standard deviation in the trait was used to calculate the weighting factor. All traits and CO_2_ concentrations were log-transformed, which improved the normality of the residuals and allowed the output to be considered as scaling slopes (Renton and Poorter 2012). We investigated both which traits responded to low CO_2_ and, for those traits that did, what the effect size of that adjustment was.

In the ANCOVA framework, the scaling slope of the trait-CO_2_ relationship then indicates the proportional change in trait value in the following way:


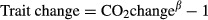
(1)

where CO_2_ change is the proportional change in CO_2_ concentration and β is the slope in the log–log plot. For example, if β were 1 then a 50% reduction in CO_2_ concentration would result in a 50% reduction in trait amount. When β is less or >1, a 50% reduction in CO_2_ will result in a less or more than 50% reduction in trait amount, respectively. A negative slope indicates an increase in trait value with a decrease in CO_2_ concentration. Subsequent to the ANCOVA analysis, differences in slope between C_3_ and C_4_ herbs and woody and herbaceous C_3_ plants were assessed by 2 sample t-tests weighted by 1/SE of the species. All statistics were performed using R, version 2.14.0 (R Foundation for Statistical Computing, Vienna, Austria). Due to the limited number of species for many traits, statistical power tended to be low; however, we judge it important enough to report those results as they reflect the current state of knowledge and to show the lack of data in important traits.

Rather than performing a meta-analysis on the available high CO_2_ experiments ourselves, we searched the literature for highly cited large-scale meta-analyses on plant traits in experimentally elevated CO_2_. From our survey, 5 large meta-analyses emerged involving tens to hundreds of plant species reporting various traits including the 6 traits that were reported for 10 or more species at reduced CO_2_ (Curtis and Wang 2001; Poorter and Navas 1988; Ainsworth and Long 2005; Ainsworth and Rogers 2005; Wang et al. 1990). From the meta-analyses, we extracted the shift in trait value at nonlimiting resources when available. We then compared this to the projected trait shift when assuming the same proportional response as to low CO_2_.

## Results

Of the 21 traits that were reported for 3 or more species, 14 showed either significant variation with CO_2_ or species response to CO_2_. Percentage values in the text below show the proportional change in trait value ± SE upon a 50% reduction in growth CO_2_ concentration (eqn 1). For each trait, species could respond to CO_2_ (adjust their trait value), show consistent variation in trait value between species over a CO_2_ gradient (species intercept or elevation of species line in trait vs. CO_2_ plot) and show significant variation in how species responded to CO_2_ (CO_2_*species interaction; Table 1). A nonsignificant interaction of species*CO_2_ for a given trait indicates that different species adjust the trait by the same proportional amount.

**Table 1:** Overview of ANCOVA results on log(trait data) versus log(CO_2_) concentration with species as covariate and as weighting factor. Traits are ordered by number of species analyzed. Slope indicates the average slope of log(trait) versus log(CO_2_) including SE. −50% CO_2_ gives the proportional change in trait given a 50% reduction in CO_2_ concentration as per *Trait change* = *CO*_*2*_ *change*^*β*^*-1* where β is the slope. Values are calculated by slope ± SE

Trait	#Species	#Studies	Slope	−50% CO_2_	p(CO_2_)	p(species)	p(CO_2_*species)
WUE (mmol mol^−1^)	26	8	0.95 ± (0.1)	−48.3% ± (3.5)	***	***	***
*A*_*net*_ (μmol·m^−2^·s^−1^)	25	15	0.7 ± (0.11)	−38.3% ± (4.5)	***	***	***
DW (g)	25	14	0.91 ± (0.16)	−46.9% ± (5.8)	***	***	ns
SLA (m^2^·g^−1^)	22	17	−0.2 ± (0.08)	+17.2% ± (6.4)	***	***	ns
*g*_*s*_ (mol·m^−2^·s^−1^)	17	11	−0.7 ± (0.13)	+59.8% ± (13.9)	**	***	**
*A*_max_ (μmol·m^−2^·s^−1^)	15	9	0.58 ± (0.09)	−33.1% ± (4.3)	*	***	ns
% Leaf *N* (g·g^−1^)	10	9	−0.2 ± (0.1)	+17.8% ± (8)	***	***	ns
PNUE (μmol·mmol·N^−1^·s^−1^)	10	2	0.22 ± (0.36)	−14.2% ± (21.6)	†	*	ns
r/s ratio	9	3	0.34 ± (0.11)	−21% ± (6.1)	***	***	**
Shoot DW (g)	7	4	0.62 ± (0.14)	−35.1% ± (6.5)	***	***	ns
% Leaf mass	5	4	−0.1 ± (0.2)	+9% ± (15)	*	***	***
Root DW (g)	5	4	1.34 ± (0.27)	−60.6% ± (7.5)	***	***	ns
Leaf DW (g)	5	4	0.83 ± (0.54)	−43.9% ± (21.5)	***	***	**
*C*_*i*_*/C*_*a*_	4	4	−0.1 ± (0.12)	+6% ± (8.8)	ns	ns	ns
Chlorophyll (μmol·g^−1^)	4	4	0.19 ± (0.09)	−12.4% ± (5.4)	ns	***	ns
Leaf *N* (g·m^−2^)	4	4	0.23 ± (0.11)	−14.8% ± (6.3)	*	ns	ns
% Stem mass	4	3	0.08 ± (0.08)	−5.4% ± (5)	ns	*	ns
Stem DW (g)	4	3	1.72 ± (0.83)	−69.7% ± (18.5)	***	***	***
# Stomata	4	2	0.08 ± (0.11)	−5.7% ± (7.3)	ns	***	ns
RuBisCO (g·m^−2^)	3	3	0.37 ± (0.14)	−22.7% ± (7.3)	ns	**	ns
Pore size (μm)	3	1	0.12 ± (0.13)	−7.8% ± (8.2)	ns	***	ns

*P*-values are ns: not significant; †<0.1, *<0.05, **<0.01, ***<0.001.

### Photosynthesis-related traits

**Figure 1:**
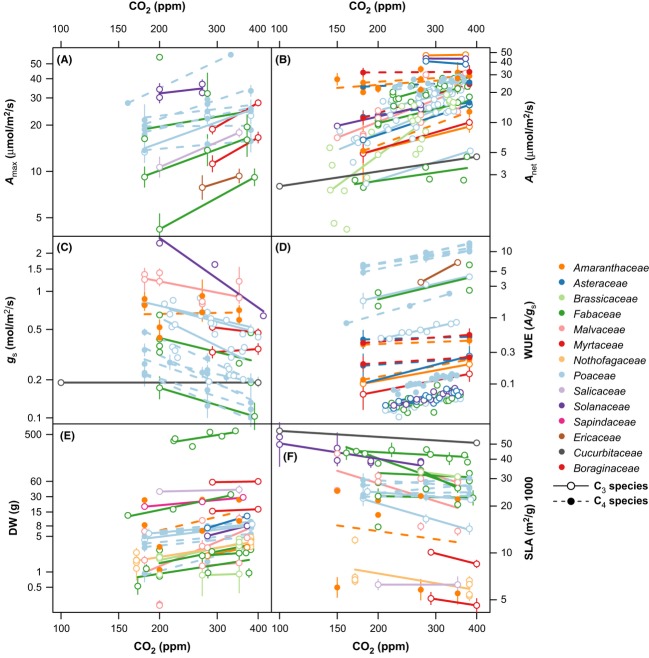
Plant trait versus growth CO_2_ concentration (note the double-log scale) of the 6 traits with the highest number of species. (A) Maximum photosynthesis (*A*_max_), (B) Net photosynthesis (*A*_net_), (C) Stomatal conductance (*g*_*s*_), (D) Intrinsic water use efficiency (net photosynthesis over *g*_*s*_, WUE), (E) Plant dry weight, (F) Specific leaf area (SLA). Each line represents the response of a single species. Open symbols: C_3_ metabolism, solid symbols: C_4_ metabolism. Error bars give SE. Different colors represent the different families the species belong to.

Across the species studied, a 50% reduction in CO_2_ did on average reduce maximum photosynthesis (*A*_max_, μmol·m^−2^·s^−1^) by 33 ± 4% (*P* < 0.05, 15 species; Fig. 1A) and net photosynthesis (*A*_net_, μmol·m^−2^·s^−1^) comparably by 38 ± 5% (*P* < 0.001, 25 species; Fig 1B). Next to this, stomatal conductance (*g*_*s,*_ mol·m^−2^·s^−1^) increased by 60 ± 14% (*P* < 0.01, 17 species; Fig. 1C). The ratio of water loss to carbon gain, intrinsic water use efficiency (*A*_net_ over *g*_*s*_, WUE), decreased by 48 ± 4% (*P* < 0.001, 26 species; Fig. 1D). For *A*_net_, WUE and *g*_s_ species showed significant variation in trait elevation (*P* < 0.001) and response to CO_2_ (interaction, *P* < 0.01). *A*_max_ however showed only significant variation in species trait elevation (*P* < 0.001).

### Growth and allocation

A halving of growth CO_2_ concentration resulted in a corresponding reduction in plant dry weight (DW) by 47 ± 6% (*P* < 0.001, 25 species; Fig. 1E). Aboveground biomass was less reduced than belowground biomass as Shoot DW was reduced by 35 ± 7% (*P* < 0.01, 7 species) and Root DW by 61 ± 8% (*P* < 0.001, 5 species). This pattern was reflected in a reduced root/shoot dry matter ratio (r/s ratio) of 21 ± 6% (*P* < 0.001, 9 species). In contrast, specific leaf area (SLA, m^2^·g^−1^) increased by 17 ± 6% (*P* < 0.001, 22 species; Fig. 1f) at reduced CO_2_. For root/shoot ratio, there was both significant variation in species trait elevation (*P* < 0.001) and response to CO_2_ (*P* < 0.01). For SLA and plant biomass, species showed significant variation in trait elevation but did not respond differently to reduced CO_2_.

### Chemical composition

Only few data on chemical composition were available, strongly limiting statistical power of our test. The concentration of chlorophyll (μmol·g^−1^, 4 species) and amount of RuBisCO (g·m^−2^, 3 species) were not significantly affected by CO_2_. Nitrogen levels in the leaf showed a contrasting response to halving CO_2_ where leaf nitrogen percent (g·g^−1^) increased by 18 ± 8 (*P* < 0.001, 10 species) whereas nitrogen content per area (g·m^−2^) decreased by 15 ± 6% (*P* < 0.05, 4 species). Neither for nitrogen per unit mass nor per unit leaf area did species have significantly different responses to reduced CO_2_.

### Differential responses of plant types

**Figure 2:**
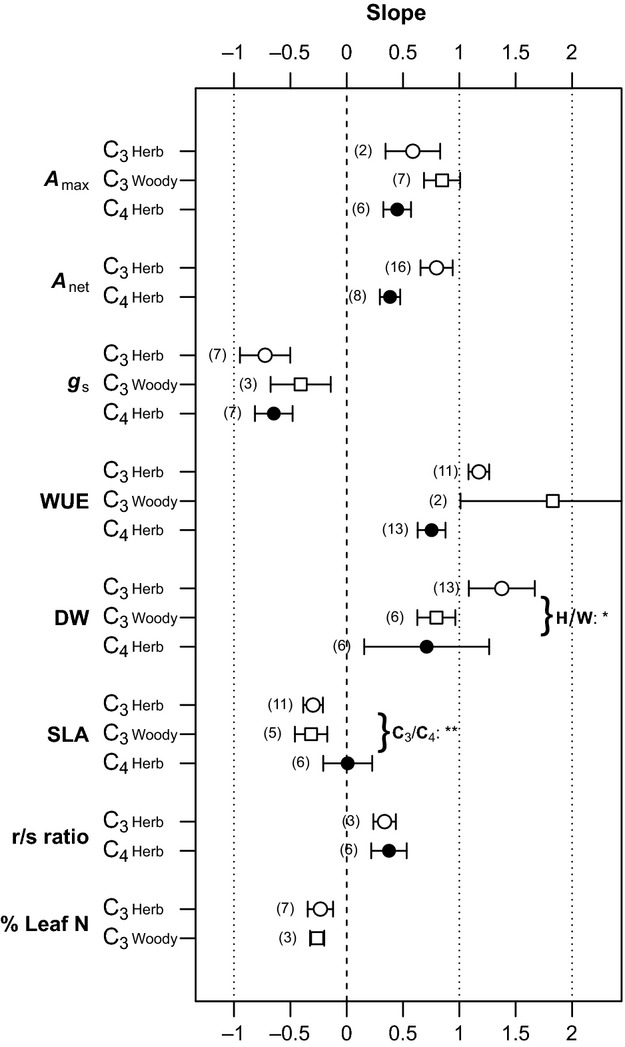
Slope of log(trait) versus log(CO_2_ concentration) for different plant types, C_3_/C_4_ and woody/herbaceous. Open circles, C_3_ herbaceous type; solid circles, C_4_ herbaceous type; open square, C_3_ woody type. ±1 indicates a 1:1 change in a trait for a change in CO_2_ concentration with a negative slope indicating an increase in trait value and a positive slope indicating a decrease in trait value. *A*_max_, maximum photosynthesis (μmol·m^−2^·s^−1^); *A*_net_, net photosynthesis (μmol·m^−2^·s^−1^); *g*_*s*_, stomatal conductance (mol·m^−2^·s^−1^); WUE, water use efficiency (mmol·mol^−1^); DW, plant dry weight (g); SLA, specific leaf area (m^2^·g^−1^); r/s ratio, root DW to shoot DW (g·g^−1^). Numbers between brackets gives the number of species for each plant type. *P*-values between plant types are *< 0.05, **< 0.01.

Due to the small number of available species, the statistical power of the comparison between plant types was limited. Thus, in a few cases, there was only a trend of differential response among C_3_, C_4_ and woody and herbaceous species. Nevertheless, interesting contrasts and similarities emerged. Figure 2 shows the contrasting slopes between plant types for the eight plant traits with nine or more species. Between C_3_ and C_4_ herbs, only the greater increase in SLA for C_3_ herbs at reduced CO_2_ was significant (*P* < 0.01). C_4_ plants showed on average a negligible SLA response to CO_2_. Net photosynthesis and dry weight seemed to be less reduced for C_4_ herbs although with small sample size and large variation this was not significant. Interestingly, plant dry weight of woody species was reduced less by 16% than that of herbaceous C_3_ plants (*P* < 0.05). Overall, for the traits shown in Figure 2, different plant types appear to show rather similar responses to reduced CO_2_.

### Comparison with elevated CO_2_ experiments

Table 2 highlights the comparison between trait responses at low CO_2_ to the response at elevated CO_2_ of the six traits that had the most data. When comparing the trait shifts found in five large meta-analyses (Curtis and Wang 2001; Poorter and Navas 1988; Ainsworth and Long 2005; Ainsworth and Rogers 2005; Wang et al. 1990) to trait shifts extrapolated from the response to low CO_2,_ a few interesting contrasts and similarities emerged. With increasing CO_2,_ the magnitude of shift in *A*_net_ deviated more from the response to low CO_2_; for *g*_*s*_, the shift was similar in magnitude to results found in FACE studies and one growth chamber meta-analysis but not another. While we found no differences between C_3_ and C_4_ plants in water use efficiency at low CO_2_, at high CO_2_ large differences are found. The increase in WUE at high CO_2_ found for C_3_ plants at FACE sites is comparable to our extrapolated response. Whole plant dry weight (DW) appears to increase much less at high CO_2_ then expected based on the low CO_2_ response. SLA, however, seems to be adjusted in a similar magnitude as expected from the response to low CO_2_.

**Table 2:** Comparison of trait shift at high CO_2_ extrapolated from low CO_2_ response (bold values) to actual changes found in three meta-analyses. If the trait adjustments are proportional from past low to future high CO_2_, the predictions from the low CO_2_ experiments should match the measured values from the high CO_2_ experiments. The measured trait shifts are from chamber studies, GC1: Curtis and Wang 2001 (700 ppm), GC2: Poorter and Navas 1988 (690 ppm), GC 3: Wang et al. 1990 (700 ppm) and FACE experiments, FACE: Ainsworth and Long 2005; Ainsworth and Rogers 2005 (560 ppm). Percentage values indicate magnitude of trait shift as compared to current, levels of CO_2_

	GC1 (700 ppm)		GC2 (690 ppm)		GC3 (700 ppm)		FACE (560 ppm)	
				
Trait	Actual	Extrapolated	Actual	Extrapolated	Actual	Extrapolated	Actual	Extrapolated
*A*_max_							+31%	**+27% ± (5)**
*A*_net_	+28%	**+63% ± (12)**	+28%	**+61% ± (12)**	+14%	**+63% ± (12)**	+26%	**+34% ± (6)**
*g*_*s*_	−11%	−**38% ± (6)**			−32%	−**38% ± (6)**	−21%	−**25% ± (4)**
WUE							+68%(C_3_) +6%(C_4_)	**+48% ± (6)**
DW	+28%	**+88% ± (21)**	+48%(C_3_) +12%(C_4_)	**+86% ± (20)**	+25%(C_3_) −3%(C_4_)	**+88% ± (21)**		
SLA			−13%	−**15% ± (5)**	−10%	−**15% ± (5)**	−6%	−**9% ± (3)**

## Discussion

This meta-analysis seeks to quantify and aggregate current knowledge on plant traits in low CO_2_. Few traits were measured for many species, and data were found for a limited number, 45, of species (Table 1). Due to the limited number of species and trait measurements, comparison between plant types, woody–herbaceous, C_3_–C_4_, was difficult. This limited data set should be taken into account when reviewing the results. Interesting results do however emerge. In response to reduced CO_2,_ plants adjusted both physiological and morphological traits (Figure 1, Table 1). The magnitude of trait adjustment varied among species for 6 of 20 traits examined (Table 1). This suggests that on order to cope with low CO_2_ different species adjust different traits. This species-level heterogeneity in response though was not clearly based on simple functional groups (Figure 2).

Below, we discuss trait shifts at low CO_2_ moving from leaf to ecosystem scales, keeping in mind that all the results discussed below are all short-time scale, plastic responses (Gerhart and Ward 1980). Plant plastic response to changing conditions occurs at different levels of organization after different periods of time (Nicotra et al. 2011).

### Leaf-level responses to low CO_2_

We found support for our hypotheses at the leaf level. We hypothesized that low CO_2_ would lead to lower photosynthetic rates (*A*), which leads to reduced growth, and that in order to partially ameliorate the photosynthetic rate reduction, plants would have higher leaf nitrogen and larger stomatal conductance (*g*_*s*_) (Sage and Reid 1995; Medlyn et al. 1999). Focussing first on *A* and *g*_s_, *A*_net_ substantially decreased at a 50% decrease in CO_2_ and *g*_*s*_ increased considerably, indicating that the increase in stomatal conductance is not enough to keep up with lower atmospheric carbon concentration.

Water use efficiency (WUE) decreased proportionally with CO_2_ (following Franks et al. 1982) but did show significant variation between species. Such a strong increase in water demand suggests great consequences for plants experiencing drought stress. However, recovery from drought at low CO_2_ was found to be similar between C_3_ (*Abutilon theophrasti*) and C_4_ (*Amaranthus retroflexus*) plants at low CO_2,_ due to less leaf loss and stomatal closure than expected for C_3_ species (Ward et al. 1995). Suggesting that there are trait shifts that mitigate some ill effects.

When comparing the magnitude of the response of *A*, *g*_*s*_ and WUE at high CO_2_ to the responses, we found at low CO_2_ a contrasting picture emerges. At low CO_2,_ no difference in WUE was found between plant types, although at high CO_2,_ C_4_ plants increase their WUE less than C_3_ plants (Poorter and Navas 1988). For C_3_ plants, the response to high CO_2_ seems similar in magnitude as to low CO_2_ for WUE. For the components of WUE, at high CO_2,_ the adjustment of *A*_net_ was greater in magnitude as to low CO_2,_ whereas the effect on *g*_*s*_ was comparable to low CO_2_, although one meta-analysis reported a far lower decrease in *g*_s_ at high CO_2_ (Table 2). The extent to which *g*_*s*_ can be reduced might be limited though. Leaf thermal regulation, which is impaired at very low stomatal conductance, could put a limit on the possible decrease. Paleo-evidence suggests this may have been relevant during a previous “rapid” transition to a high CO_2_ atmosphere at the Triassic–Jurassic boundary (Mcelwain et al. 1992).

At the level of enzymes and leaf chemical composition, we found no evidence that RuBisCO content and chlorophyll content were affected by reduced CO_2;_ the power of our test was limited because few studies measured those traits (Table 1). Leaf nitrogen (N) content per mass did increase but N content per area decreased in response to low CO_2_; this contrasting response might be explained by the higher specific leaf area (SLA) in low CO_2_. A higher SLA indicates lower nitrogen containing mass per area. Whether or not the nitrogen increase per mass is the result of an increase in certain nitrogen-rich chemical compounds or a decrease in carbon-rich compounds, as carbon is a limiting resource, is unclear. Clearly more work is needed on the enzymatic and chemical response of plants to low CO_2_.

Plants adjust their leaf morphology in order to cope with low concentrations of atmospheric CO_2_. At half of ambient CO_2,_ leaf SLA increased by 15%. This increase has two potential advantages to the plant: first, when CO_2_ is limiting, more leaf area per unit C invested in leaves allows for lower carbon costs per unit carbon capture; second, at the leaf level, higher SLA leaves may improve mesophyll conductance (Vitousek et al. 2001). While increased *g*_*s*_ at low CO_2_ can maintain internal CO_2_ concentration up to a point, at low CO_2_ concentrations the diffusion of CO_2_ inside the leaf can become limiting to photosynthesis as well (Keenan et al. 2009). Higher SLA indicates either thinner or less dense leaves with more internal air space, which in many cases leads to greater mesophyll conductance to CO_2_ (Loreto et al. 2011). However, a higher SLA could also be a result of less starch or less other nonstructural carbohydrate present in the leaf at low CO_2_ concentrations (Poorter et al. 1993). It is interesting to find that at high CO_2,_ SLA follows the same trend as at low CO_2_. The relative contributions of the above factors to reducing and increasing SLA are an interesting avenue to pursue further.

### Plant-level responses to low CO_2_

While plants adjust their gas exchange and leaf morphology in response to reduced CO_2,_ photosynthetic rates are nevertheless reduced, resulting in less biomass but also shifts in allocation between root and shoot. Plant biomass (g dry weight) decreases proportionally at a 50% CO_2_ reduction, which is more pronounced in belowground biomass as is illustrated by a reduction in root-to-shoot ratio. This could be the result of plants balancing their nutrient gain and their carbon gain to the now more limiting carbon resource (Bloom et al. 2012; Chapin et al. 1993) or some specific source-sink relationship between root and shoot, that is, fixed in the plant's metabolism, similar to the idea of a fixed *c*_*i*_*/c*_*a*_ ratio for species across time (Ehleringer and Cerling 2008; Gerhart et al. 2013; Franks et al. 1982). At low CO_2,_ photosynthesis per area is lower, so the amount of sugars available for the roots is less per unit of shoot biomass. This shift in allocation at low CO_2_ may have important implications for species interactions, particularly in tree–savanna grass interactions (Bond and Midgley 2012): at low CO_2,_ the regrowth capacity following disturbance of tree species is much diminished, adding weight to the importance of fire and herbivory as ecosystem shaping factors (Kgope et al. 2010; Bond and Midgley 2012). Next to allocation, the increased SLA at low CO_2_ might lead to greater food availability and thus pressure from herbivores as high SLA leaves are eaten more readily (Poorter et al. 1993). Faster decomposition rates and nutrient cycling of high SLA leaves would also have large ecosystem effects by allowing faster nutrient cycling (Cornelissen et al. 2006).

### Plant types, growth environments, and low CO_2_ response

Differences between C_3_, C_4_, woody, and herbaceous species were not as pronounced as hypothesized. Results suggested differences between plant types although the responses of only few traits were significantly different. C_3_ herbs had a significantly greater increase in SLA than C_4_ herbs, which on average showed no response. Decreases in biomass diminished starting from herbaceous C_3_, woody C_3_ to herbaceous C_4_. However, only the difference between woody and herbaceous C_3_ was significant with woody plants having a smaller reduction in biomass at low CO_2_. This result mirrors that at high CO_2_ experiments where increased CO_2_ generally leads to a greater relative growth rate (RGR) increase for fast growing, herbaceous, plants than slow growing, woody, plants (Poorter and Navas 1988). As fast growers “win” more at high CO_2_ they “lose” more at low CO_2_. While the smaller decrease in biomass for herbaceous C_4_ than for C_3_ is not significant, it should be noted that the average reduction in *A*_net_ and *A*_max_ is also lower than in C_3_ herbs. This may point to a smaller reduction in biomass accumulation for C_4_ herbs via a smaller reduction in photosynthesis. With more data on woody and C_4_ species, such differences between plant types and their underlying mechanism may become more apparent.

The results presented here summarize the effects of low CO_2_ at high water and high nutrients. One important caveat to consider, however, is that that there are many potential interactions between CO_2_, water, and nutrients as is shown by some studies. Low nutrients in the form of low P limited photosynthetic rates even further at low CO_2_ in *Lupinus albus* (Campbell and Sage 2012) and *Populus deltoides* (Lewis et al. 2012). C_3_ (*Abutilon theophrasti*) and C_4_ (*Amaranthus retroflexus*) plants recovered similarly from drougth at low CO_2_ (Ward et al. 1995). This shows that other environmental factors strongly influence the effect of CO_2_ on plant traits. However, most data were available for well-watered, high-nutrient growth experiments. The interactions between CO_2_, light, nutrients and water are clearly important when extrapolating from growth chamber experiments to glacial environments, but a full understanding of the interactions would require many more or more extensive studies.

Experimental results presented here on plastic responses need to be put into context with other sources of information on plant traits in the past including measurements on paleomaterials. In the past, CO_2_ has proven to be a strong selective agent altering worldwide floristic composition (McElwain et al. 1993). Thus, evolution and selection have likely occurred with increasing CO_2,_ and it is therefore important to determine whether the traits of modern plants grown under low CO_2_ compare to the traits of plants that lived in a low CO_2_ atmosphere. The regeneration of *Silene stenophylla* buried in Siberian permafrost for over 30 ka (Yashina et al. 2012) provides an interesting opportunity for testing the response of modern plants and ancient plants to low CO_2_ and how similar they are. It is also likely that in the period since the low CO_2_ in the Last Glacial Maximum (LGM), species with short generation turnover have evolved more compared with long generation turnover species, although the drastic increase from 280 to 700 pmm within 250 years (1850–2100) will likely constrain the values to which traits have been adjusted. A full understanding of plant response to the transition from the LGM to current and future CO_2_ levels must include both evolutionary adaptation and plastic responses.

## Conclusion

In conclusion, we found that, despite the more limited set of low CO_2_ studies compared with high CO_2_ studies, a general response is emerging. Plant response to reduced atmospheric CO_2_ involves a complex suite of trait adjustments. In order to diminish the effects of reduced CO_2_ plants open their stomata wider, invest more in aboveground biomass and increase their SLA. Despite these adjustments, photosynthetic rate is nevertheless reduced, leading to a proportional reduction in biomass accumulation. Both trait adjustment and growth effect varies among species, but this variation does not appear to be a function of simple plant functional groups. Trait adjustments at low CO_2_ as compared to high CO_2_ were proportionally similar for *g*_*s*_, WUE and SLA but responses at low CO_2_ were greater than proportional for *A*_*net*_ and biomass. In other words, the data suggest that in terms of water relations and leaf morphology, the responses to low and high CO_2_ are proportional and opposite. Carbon gain and whole plant growth rate are more complex—responses to low CO_2_ in these cases are more extreme. At high CO_2,_ other factors such as nutrient and light availability could control these traits. To understand the response of plants to future high CO_2,_ it is important to understand how and when other factors become drivers for certain traits. Our understanding of plant response to CO_2_ benefits from data from both low and high CO_2_ conditions. The shape of that response will become increasingly relevant in a high CO_2_ future.

## Supporting Information

**Table S1.** Overview of trait measurements reported for three or more species. **V**: Traits reported with error value, **X**: Traits reported without SE. Traits are ordered from most to least measured in low CO_2_ studies. SLA/LMA, specific leaf area or recalculated leaf mass per area (g·m^−2^); *g*_*s*_, stomatal conductance (mol·m^−2^·s^−1^); DW, plant dry weight (g); *A*_max_, maximum photosynthesis (μmol·m^−2^·s^−1^); *A*_net_, net photosynthesis (μmol·m^−2^·s^−1^); WUE, water use efficiency (mmol·mol^−1^); PNUE, photosynthetic nitrogen use efficiency (μmol·mmol·N^−1^·s^−1^); r/s ratio, root-to-shoot ratio DW; C_i_/C_a_, ratio intercellular to atmospheric CO_2_; % Leaf *N*, percentage *N* per weight; % Leaf mass, percentage leaf DW of total DW; Root DW, root dry weight (g); Leaf DW, leaf dry weight (g); Chlorophyll, chlorophyll content (μmol·g^−1^); Leaf *N*, N per area (g·m^−2^); %Stem mass, percentage stem mass of total weight; RuBisCO, RuBisCO content (g·m^−2^); Shoot DW, shoot dry weight (g); %Root DW, percentage root DW of total DW; Pore size, diameter of stomatal pore (μm).

**Data S1.** Meta-analysis data sheet compiled and used for this paper.

**Data S2.** Column headers used in the meta-data file with their description and meaning.
