# Supplementary material for: Meta-analysis reveals profound responses of plant traits to glacial CO2 levels
Source: Ecol Evol. 2013 Oct 18;3(13):4525–35. doi: 10.1002/ece3.836 (PMC3856751; doi:10.1002/ece3.836)
Supplement: Supplementary file 2 [file ece30003-4525-SD2.docx]

Agüera E, Ruano D, Cabello P, de la Haba P (2006) Impact of atmospheric CO2 on growth, photosynthesis and nitrogen metabolism in cucumber (Cucumis sativus L.) plants. *Journal of plant physiology*, **163**, 809–17.

Allen LH, Bisbal EC, Boote KJ, Jones PH (1991) Soybean Dry Matter Allocation under Subambient and Superambient Levels of Carbon Dioxide. *Journal of Agronomy*, 875–883.

Baker JT, Allen LH, Boote KJ, Jones P, Jones JW (1990) Rice Photosynthesis and Evapotranspiration in Subambient, Ambient, and Superambient Carbon Dioxide Concentrations. *Journal of Agronomy*, **840**, 834–840.

Bunce JA (2011) Are Annual Plants Adapted to the Current Atmospheric Concentration of Carbon Dioxide? *International Journal of Plant Sciences*, **162**, 1261–1266.

Campbell CD, Sage RF (2006) Interactions between the effects of atmospheric CO2 content and P nutrition on photosynthesis in white lupin (Lupinus albus L.). *Plant, Cell and Environment*, **29**, 844–853.

Campbell CD, Sage RF, Kocacinar F, Way D a. (2005) Estimation of the whole-plant CO2 compensation point of tobacco (Nicotiana tabacum L.). *Global Change Biology*, 050922094851001–???

Cowling SA, Sage RF (1998) Interactive effects of low atmospheric CO2 and elevated temperature on growth, photosynthesis and respiration in Phaseolus vulgaris. *Plant, Cell and Environment*, **21**, 427–435.

Cunniff J, Osborne CP, Ripley BS, Charles M, Jones G (2008) Response of wild C 4 crop progenitors to subambient CO 2 highlights a possible role in the origin of agriculture. *Global Change Biology*, **14**, 576–587.

Dippery JK, Tissue DT, Thomas R., Strain BR (1995) Effects of low and elevated CO2 on C3 and C4 annuals I. Growth and biomass allocation. *Oecologia*, 13–20.

Ghannoum O, Phillips NG, Conroy JP *et al.* (2010) Exposure to preindustrial, current and future atmospheric CO2 and temperature differentially affects growth and photosynthesis in Eucalyptus. *Global Change Biology*, **16**, 303–319.

Gonzàlez-Meler MA, Blanc-Betes E, Flower CE, Ward JK, Gomez-Casanovas N (2009) Plastic and adaptive responses of plant respiration to changes in atmospheric CO(2) concentration. *Physiologia plantarum*, **137**, 473–84.

Hovenden MJ, Schimanski LJ (2000) Genotypic differences in growth and stomatal morphology of Southern Beech, Nothofagus cunninghamii, exposed to depleted CO2 concentrations. *Australian Journal of Plant Physiology*, **27**, 281–287.

Kgope BS, Bond WJ, Midgley GF (2010) Growth responses of African savanna trees implicate atmospheric [CO2] as a driver of past and current changes in savanna tree cover. *Austral Ecology*, **35**, 451–463.

Lewis JD, Ward JK, Tissue DT (2010) Phosphorus supply drives nonlinear responses of cottonwood (Populus deltoides) to increases in CO2 concentration from glacial to future concentrations. *The New phytologist*, **187**, 438–48.

Luo Y, Sims DA, Griffin KL (1998) Nonlinearity of photosynthetic responses to growth in rising atmospheric CO 2 : an experimental and modelling. *Global Change Biology*, **4**, 173–183.

Maherali H, Reid CD, Polley HW, Johnson HB, Jackson RB (2002) Stomatal acclimation over a subambient to elevated CO2 gradient in a C3/C4 grassland. *Plant, Cell and Environment*, **25**, 557–566.

Mohan J., Clark J., Schlesinger W. (2004) Genetic variation in germination , growth , and survivorship of red maple in response to subambient through elevated atmospheric CO 2. *Global change biology*, **10**, 233–247.

Overdieck D (1989) The effects of preindustrial and predicted future atmospheric CO2 concentration on Lyonia mariana L.D. Don. *Functional Ecology*, **3**, 569–576.

Overdieck D, Reid C, Strain BR (1988) The Effects of Preindustrial and Future CO2 Concentrations on Growth, Dry Matter Production and the C/N Relationship in Plants at Low Nutrient Supply. *Angewandte Botanik*, **62**, 119–134.

Polley WH, Johnson HB, Mayeux HS (1992) Growth and Gas Exchange of Oats (Avena sativa) and Wild Mustard (Brassica kaber) at Subambient CO2 Concentrations. *International Journal of Plant Sciences*, **153**, 453–461.

Polley WH, Johnson HB, Mayeux HS, Malone SR (1993) Physiology and Growth of Wheat Across a Subambient Carbon Dioxide Gradient. *Annals of Botany*, **71**, 347–356.

Polley WH, Johnson HB, Mayeux HS (1994) Increasing CO2 : Comparative Responses of the C4 Grass Schizachyrium and Grassland Invader Prosopis. *Ecology*, **75**, 976–988.

Polley HW, Johnson HB, Mayeux HS (1995) Nitrogen and Water Requirements of C3 Plants Grown at Glacial to Present Carbon Dioxide Concentrations. *Functional Ecology*, **9**, 86–96.

Polley HW, Johnson HB, Derner JD (2002) Soil- and plant-water dynamics in a C3/C4 grassland exposed to a subambient to superambient CO2 gradient. *Global Change Biology*, **8**, 1118–1129.

Possell M, Nicholas Hewitt C (2009) Gas exchange and photosynthetic performance of the tropical tree Acacia nigrescens when grown in different CO(2) concentrations. *Planta*, **229**, 837–46.

Possell M, Nicholas Hewitt C, Beerling DJ (2005) The effects of glacial atmospheric CO2 concentrations and climate on isoprene emissions by vascular plants. *Global Change Biology*, **11**, 60–69.

Sage RF, Reid CD (1992) Photosynthetic acclimation to sub-ambient CO2 (20 Pa) in the C3 annual Phaseolus vulgaris L. *Photosynthetica*, **27**, 605–617.

Tissue DT, Griffin KL, Thomas R., Strain BR (1995) Effects of low and elevated CO2 on C3 and C4 annuals II. Photosynthesis and leaf biochemistry. *Oecologia*, 21–28.

Tonsor SJ, Scheiner SM (2007) Plastic trait integration across a CO2 gradient in Arabidopsis thaliana. *The American naturalist*, **169**, E119–40.

Vogan PJ, Sage RF (2012) Effects of low atmospheric CO2 and elevated temperature during growth on the gas exchange responses of C3, C3-C4 intermediate, and C4 species from three evolutionary lineages of C4 photosynthesis. *Oecologia*, **169**, 341–52.

Ward JK, Strain BR (1997) Effects of low and elevated CO2 partial pressure on growth and reproduction of Arabidopsis ttiaiiana from different elevations. *Plant, Cell and Environment*, **20**, 254–260.

Ward JK, Tissue DT, Thomas RB, Strain BR (1999) Comperative responses of model C3 and C4 plants to drought in low and elevated CO2. *Global Change Biology*, **5**, 857–867.

Ward JK, Myers D a, Thomas RB (2008) Physiological and growth responses of C3 and C4 plants to reduced temperature when grown at low CO2 of the last ice age. *Journal of integrative plant biology*, **50**, 1388–95.
