# Supplementary material for: Meta-analysis reveals profound responses of plant traits to glacial CO2 levels
Source: Ecol Evol. 2013 Oct 18;3(13):4525–35. doi: 10.1002/ece3.836 (PMC3856751; doi:10.1002/ece3.836)
Supplement: Supplementary file 3 [file ece30003-4525-SD3.pdf]

Supplement 3. Column headers used in the meta-data file with their description and meaning.

Column header	***Description***
**reference**	Reference from which the data values were obtained
**pubyear**	Publication year of reference
**genus**	Genus name of species
**species**	Species name of species
**name**	Combined genus and species name
**c3c4**	Indicates whether a species is C3 or C4
**woodherb**	Indicates whether a species is woody or herbaceous
**genotype**	Gives the genotype of the species analysed, when reported
**elevation**	Gives the elevation of the experiment, when reported
**treatment**	Method of treatment: Climate room, Greenhouse, Mayeux*et al* tube system, or selection experiment in climate room
**soilaqua**	Indicates soil type or aquatic medium used to grow the plants
**samplesize**	Number of individuals measured
**duration**	The CO_2 treatment duration
**germination**	Indicates whether plants were germinated under growth conditions or previous to the experimental period (possibly from a cutting)
**co2conc**	Concentration to which plants were exposed
**humidity**	Humidity levels during the experiment
**potsize**	Size of pots used (in litters)
**plantsm2**	Density of plants grown
**minppfd**	Minimum light level during experiment
**lightlevelav**	Average light level during experiment
**maxppfd**	Maximum light level during experiment
**fertelization**	Fertilization level
**tempday**	Day temperature
**tempnight**	Night temperature
**daylength**	Hours of light during each day
**watering**	Watering level during the experiment
ertyp	Denotes the type of error reported, Standard error (SE) or Standard Deviation (SD)
graph	Indicates whether a data value comes from a graph
error	Gives the error for the data value
**rootw**	Root weight (g dry weight)
**ratrs**	ratio root weight to shoot weight
**shootw**	Shoot weight (g dryweight)
**stemw**	Stem weight (g dryweight)
**stempcnt**	Percentage stem weight of total dry weight
**leafw**	Leaf weight (g dryweight)
**leafpcnt**	Percentage leaf weight of total dry weight
**totw**	Total plant dry weight (g)
**growth**	Growth ($g\ m^{-2}\ d^{-1}$)
**lma**	Leaf mass per area ($g\ m^{-2}$)
**sla**	Specific leaf area ($m^2\ g^{-1}$)
**nrstom**	Number of stomata per mm^2
**stominx**	Stomatal index
**poresize**	Stomatal pore size (µm)
**gs**	Stomatal conductance ($mol\ m^{-2}\ g^{-1}$)
**cica**	Ratio of internal to external CO_2
**amx**	Maximum photosynthesis ($\mu mol\ m^{-2}\ g^{-1}$)
**ant**	Net photosynthesis ($\mu mol\ m^{-2}\ g^{-1}$)
**wue**	Water use efficiency ($mmol\ mol^{-1}$)
**chloro**	Amount of chlorophyll ($\mu mol\ g^{-1}$)
**rubisco**	Amount of RuBisCO ($g\ m^{-2}$)
**leafn**	Amount of leaf *N* ($g\ m^{-2}$)
**leafnpcnt**	Percentage leaf *N* ($g\ g^{-1}$)
